# Qigong Exercise in Frailty, Psychological Health, and Spiritual Well-Being of Middle-Aged and Older Blacks

**DOI:** 10.1093/geroni/igaa057.575

**Published:** 2020-12-16

**Authors:** Pei-Shiun Chang, Yvonne Lu, Susan Ofner, Chi Nguyen

**Affiliations:** 1 Indiana University at Bloomington, Bloomington, Indiana, United States; 2 Indiana University, Indianapolis, Indiana, United States; 3 Indiana University, Bloomington, Indiana, United States

## Abstract

Interventions are needed to address the frailty and psychological health in middle-aged and older African Americans. Qigong, a traditional medicine exercise, consists of gentle body movements, breathing exercise, and meditation. The benefits of Qigong exercise have been widely reported in Asian adults but there have been no known studies testing Qigong exercise in the African American population. The purpose of this pilot study was to evaluate the potential benefits of an 8-week Qigong exercise in physical and functional ability, balance, frailty, depression and anxiety, and spiritual well-being in community-dwelling middle-aged African Americans using a single group design. Fifteen African Americans aged 45 to 85 years were recruited to receive Qigong exercise over 16 bi-weekly, one-hour sessions. The Layers Module that unfolds the complex multidimensional benefits of Qigong exercise on physical, mental, and spiritual dimensions was used to guide the outcome measures. Data were collected at baseline and 14 days post intervention. Results showed positive trends in repeat chair stands, physical function, and spiritual well-being (p<0.05) with effect sizes ranging from 0.45 to 0.87. Despite no significance, over 52% of the participants showed improved depression scores, fast gait speed, and standing balance than baseline. Nearly 42% demonstrated some levels of frailty improvement than the baseline. No adverse events related to Qigong exercise intervention was reported. Qigong exercise is a potentially promising intervention which needs further testing in a randomized clinical trial.

